# HaTU-Net: Harmonic Attention Network for Automated Ovarian Ultrasound Quantification in Assisted Pregnancy

**DOI:** 10.3390/diagnostics12123213

**Published:** 2022-12-18

**Authors:** Vivek Kumar Singh, Elham Yousef Kalafi, Eugene Cheah, Shuhang Wang, Jingchao Wang, Arinc Ozturk, Qian Li, Yonina C. Eldar, Anthony E. Samir, Viksit Kumar

**Affiliations:** 1 Center for Ultrasound Research & Translation at the Massachusetts General Hospital, Department of Radiology, Harvard Medical School, Boston, MA 02114, USA; 2 Department of Ultrasound, The Third Hospital of Hebei Medical University, Shijiazhuang 050051, China; 3 Faculty of Mathematics and Computer Science, Weizmann Institute of Science, Rehovot 7610001, Israel

**Keywords:** follicle monitoring, deep learning, antral follicle count, harmonic attention, ultrasound imaging, pelvic ultrasound

## Abstract

Antral follicle Count (AFC) is a non-invasive biomarker used to assess ovarian reserves through transvaginal ultrasound (TVUS) imaging. Antral follicles’ diameter is usually in the range of 2–10 mm. The primary aim of ovarian reserve monitoring is to measure the size of ovarian follicles and the number of antral follicles. Manual follicle measurement is inhibited by operator time, expertise and the subjectivity of delineating the two axes of the follicles. This necessitates an automated framework capable of quantifying follicle size and count in a clinical setting. This paper proposes a novel Harmonic Attention-based U-Net network, HaTU-Net, to precisely segment the ovary and follicles in ultrasound images. We replace the standard convolution operation with a harmonic block that convolves the features with a window-based discrete cosine transform (DCT). Additionally, we proposed a harmonic attention mechanism that helps to promote the extraction of rich features. The suggested technique allows for capturing the most relevant features, such as boundaries, shape, and textural patterns, in the presence of various noise sources (i.e., shadows, poor contrast between tissues, and speckle noise). We evaluated the proposed model on our in-house private dataset of 197 patients undergoing TransVaginal UltraSound (TVUS) exam. The experimental results on an independent test set confirm that HaTU-Net achieved a Dice coefficient score of 90% for ovaries and 81% for antral follicles, an improvement of 2% and 10%, respectively, when compared to a standard U-Net. Further, we accurately measure the follicle size, yielding the recall, and precision rates of 91.01% and 76.49%, respectively.

## 1. Introduction

Ovarian reserve, defined as the total number of ovarian follicles, indicates the quality and quantity of the primordial follicular pool in the ovaries [1]. Patients with infertility have shown a correlation between predictors of functional ovarian reserves and ovarian responses to pregnancy outcomes [2]. Antral follicle count (AFC) and size, obtained using TransVaginal UltraSound (TVUS) images, are non-invasive imaging biomarkers used to assess and quantify ovarian reserve [2,3].

The primary aim of ovarian reserve monitoring is to measure the number and size of ovarian follicles and the number of antral follicles, which are, on average, 2–10 mm in diameter [4]. Follicle size is measured by taking the average of each follicle’s two largest orthogonal diameters [5]. There are limitations to manually estimating the size and count of follicles; the process is time-consuming, inconsistent [4], and highly variable depending upon the actual shape of primarily non-spherical follicles [3]. An accurate, automated method to segment ovaries and follicles and count the follicles could optimize the clinical flow and reduce subjectivity.

**Figure 1:**
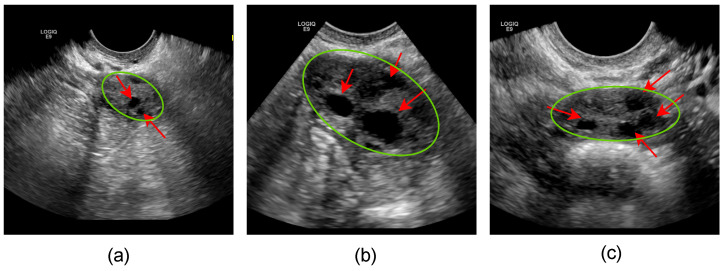
Illustration of three examples from the ovary dataset. Here, examples (**a**–**c**) show the ovary and follicles highlighted by the green circle and red arrows respectively.

Developing an automated solution for ovary and follicle segmentation incorporates numerous challenges. Figure 1 shows three examples of US images of ovary and follicles. Quantifying ultrasound images ensures reproducibility and reliability [6,7]. Ultrasound imaging artifacts impede the performance of deep learning-based segmentation methods. Blurred ambiguous boundaries further compound challenges in delineating tissue boundaries and the presence of acoustic shadowing [8]. Many image processing and computer vision-based methods are suggested to overcome these challenges that involve geometric features [9] and watershed [10]. Active contours-based [11] approaches have been used to segment the ovary and follicles. Traditional ovary and follicle monitoring methods have been frequently explored with large and distinctly visible follicles [3,12]. Boundary ambiguity is noticeable in ovarian and follicular images. The traditional methods have some limitations, such as watershed or thresholding approaches generating discontinuities and variances of intensity in the ovarian ultrasound images. Their slow speed creates challenges to adopt in actual practice clinical settings.

Convolutional neural networks (CNNs) have shown substantial performance, and accuracy advancements over conventional methods [13]. With the great success of CNNs, multiple popular segmentation methods have been developed such as FCN [14], U-Net [15], SegNet [16], Attention-UNet [17], DeepLabv3+ [18], ERFNet [19], and BiseNetv2 [20] that segment the objects or anatomies. These methods achieved state-of-the-art results for various semantic segmentation tasks.

Recently, many deep learning-based methods have been developed for analyzing medical images [21,22]. Specifically, U-Net-based models have achieved great success with medical image segmentation [23,24,25]. Meng et al. [26] proposed an instinctive deep learning-based contour regression model for biomedical image segmentation. The authors aggregated multi-level and multi-stage networks to regress the contour coordinates in an end-to-end manner rather than pixel-wise dense predictions. The authors used this method to segment the fetal head in ultrasound images and the optic disc and optic cup in color fundus images. Valanarasu et al. [27] presented a network architecture called KiU-Net, which projects data onto higher dimensions and picks finer details when compared to a standard U-Net. The suggested method addressed the performance failures when segmenting smaller anatomical structures with blurred, noisy boundaries. The authors performed the brain anatomy segmentation from 2D ultrasound (US). Singh et al. [28] proposed an automated solution to segment the breast lesion from the US images. The recommended method used generative adversarial learning (GAN) networks. The introduced method efficiently extracts spatial features such as texture, edge, shape, intensity, and global information. The authors used an attention mechanism that highlights the most relevant features and ignores the background ones. However, the GAN-based method has limitations due to its computational complexity and fails to delineate if the lesion shape is not complete. Further, Yang et al. [29] incorporated the multi-directional recurrent neural network (RNN) with a customized CNN to extract spatial intensity concurrencies to eliminate boundary ambiguities. The author employed semantic segmentation methods in prenatal ultrasound volumes that potentially encourage fetal health monitoring.

Various deep learning-based methods have been used to detect and segment ovary, and antral follicles [8,30]. Li, Haoming et al. [8] proposed an ovary and follicle segmentation model called CR-UNet, consisting of spatially recurrent neural networks incorporated into a standard U-Net. The recommended network has limitations in correctly delineating and detecting the follicles that are joined with each other [30]. Gupta et al. [31] developed a deep learning-based framework for ovarian volume computation that utilizes 3D US volumes and the axial orientation. The authors evaluated their methods on 20 3D ovarian US volumes that enhanced the grade of the 3D rendering of the ovary and addressed the issue of combined follicles in segmentation. Yang et al. [32] introduced ovary and follicles segmentation using the contrastive rendering (C-Rend) framework. The authors employed the semi-supervised learning approach with C-Rend leveraging unlabeled 3D ultrasound for better performance. However, this study has some limitations during inference due to its hyperparameter default value which might not be the most suitable setting for each 3D US.

**Figure 2:**
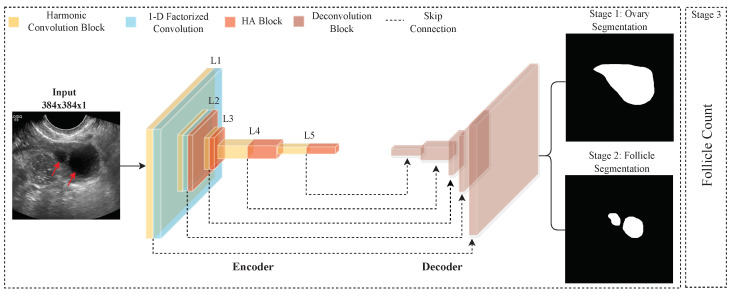
A general framework of ovarian ultrasound quantification. Here $L_{n}$ refers to the number of layers (i.e., 1 to 5) in the encoder network.

The clinical need to monitor the smaller follicles automatically and precisely, such as antral follicles (follicles that are 2–8 mm in average diameter) [4] could not be met using the current AI segmentation technologies, due to some limitations, such as deep learning models overfitting and imaging artifacts. Therefore, the main aim of this paper is to develop an automated method for efficient ovary and follicles segmentation in ovarian TVUS images to facilitate measuring the size of the follicle. Figure 2 shows the schematic view of our proposed framework. The framework incorporates three stages, i.e., ovary segmentation, follicle segmentation, and follicle counting. We designed a new segmentation method that replaces the standard 2D convolution layer with a harmonic convolution. In contrast, [33] harmonic convolution combines the learned kernels with predefined filters for feature learning. This weighted combination reduces overfitting and computational complexity. The proposed HaTU-Net method effectively extracts the features that allow precise segmentation of the ovary and follicles from the US images. Moreover, we developed a new attention block that helps to improve the segmentation performance by encouraging the feature discriminability between the pixels and ignoring US imaging artifacts. In summary, our major contributions are in four folds:

- We propose a segmentation network called HaTU-Net to segment ovaries and follicles from TVUS images.
- We propose using harmonic convolution [33] to replace the standard convolutional filter. The input image is first decomposed using the discrete cosine transform (DCT); these transformed signals are combined using learned weights.
- We developed harmonic attention (HA) block to improve feature discriminability between the target and background pixels in the segmentation stage. The HA block encourages the features by avoiding the artifacts, and support for the HaTU-Net leads to improved segmentation results.
- Our experimental results confirm HaTU-Net has shown significant improvement compared to the various state-of-the-art segmentation methods (U-Net [15], AttentionU-Net [17], R2U-Net [34], U-Net++ [35], and DeepLabv3+ [18]).

The remainder of this paper is organized as follows: Section 2 describes the dataset and methodology. Section 3 explains our experimental results and highlights the limitations of the work. Section 4 completes our study and suggests some future lines of research.

## 2. Material and Methods

This section presents a detailed description of the ovary US dataset and architecture details of the proposed HaTU-Net as depicted in Figure 2. The proposed method incorporated two main parts: harmonic convolution and harmonic attention (HA) blocks.

### 2.1. Dataset

The Institutional Review Board approved the retrospective study (IRB), and the requirement for informed consent was waived. The radiology reports of adult patients who underwent transvaginal ultrasound (TVUS) exams between 2005 and 2019 in a single institution were reviewed, and a total of 197 eligible patients were identified and selected. The inclusion criteria were: (1) premenopausal female adult patients, (2) underwent TVUS exams, (3) normal ovary on the pelvic ultrasound, and (4) available B-mode static images of the ovary. Patients with the following criteria were excluded: (1) low-quality US images with obscure boundaries of the ovary, (2) abnormal ovary findings on the ultrasound, or (3) known ovarian pathologies or ovarian surgical history.

All TVUS exams were performed using GE LOGIQ E9 (General Electric Healthcare, Waukesha, WI, USA) ultrasonic system equipped with a transvaginal IC5-9 transducer. US exams were reviewed in a picture archiving and communication system (PACS) to extract the DICOM images of ovaries in sagittal and coronal planes. Using the MicroDicom Viewer tool (Version 3.2.7, Sofia, Bulgaria), a radiologist who had ten years of experience in pelvic ultrasound annotated the contours of the ovary and follicles following a specific protocol, (1) annotated all follicles of 2–28 mm [36] in diameter within each ovary, (2) annotate ovary and follicles in different colors, and (3) avoid overlaps among annotations within the same ovary. Another senior radiologist with more than ten years of experience reviewed the annotated images as part of the quality control process. Table 1 shows the overview of the ovarian dataset totaling 767 images with qualified annotations, split into training, validation, and testing sets with 466, 160, and 141 TVUS images, respectively.

### 2.2. HaTU-Net Architecture

Figure 2 illustrates the general framework of the proposed ovarian ultrasound quantification. This includes the three stages: ovary segmentation, follicles segmentation, and follicle count. Note that HA refers to harmonic attention block, and red arrows show the follicles. We propose the ovary and follicles segmentation model called HaTU-Net. It consists of encoder and decoder networks. Each network consists of five layers with added skip connections. The encoder network utilized a harmonic convolutional layer with kernel size 3×3 instead of standard 2D convolutional layers. Each layer within the encoder employs batch normalization followed by the ReLU activation function. The first encoder layer combines harmonic convolution with a 1-D kernel factorization [19], allowing feature extraction with low computational costs. The second layer uses a variety of harmonic convolution, 1-D kernel factorization, and harmonic attention (HA) blocks. The attention mechanism boosts the feature discriminability between the target and background pixels. The last three encoder layers use a harmonic convolution with an HA block to enable channel interdependencies, and highlight features specific to the ovaries and follicles. After each layer, dimensionality reduction is achieved using a max-pooling operation with a kernel size of 2×2. In the decoder, feature upsampling is performed through the *conv-transpose2D* operation. Each encoder layer’s features are concatenated with the corresponding features in the decoding layer (skip connections). A threshold of 0.5 is used to generate the final predicted mask for ovary and follicles segmentation.

### 2.3. Feature Extraction with Harmonic Convolution

Motivated by [33], we replaced the standard convolution with a harmonic convolution, i.e., a weighted sum of the responses to a discrete cosine transform (DCT) filter bank to pull the harmonics from lower-level features to decrease the burden of overfitting. The DCT is a separable transform that converts a signal from the temporal domain to the spectral domain. The DCT of a 2D image *I* of size H×W with a one-pixel discretization step can be formulated as follows [33]:

(1) $$T_{u,v}=\sum_{i=0}^{H-1}\sum_{j=0}^{W-1}\sqrt{\frac{\beta_{u}}{H}}\sqrt{\frac{\beta_{v}}{W}}I_{\left(i,j\right)}\times \cos\left[\frac{\pi }{H}\left(i+\frac{1}{2}\right)u\right]\cos\left[\frac{\pi }{W}\left(j+\frac{1}{2}\right)v\right],$$

where $T_{u,v}$ is the coefficient corresponding to a sinusoidal frequency of *u* and *v* in the two orthogonal directions. Here $\beta_{0}$ = 1 and $\beta_{u}$ = 2 are scaling factors used to normalize the value of the basis function.

The input image features are decomposed using the DCT transform to perform the convolution operation. A kernel size of f×f and depth of $f^{2}$ in the filter bank is used. Let $\gamma_{\left(u,v\right)}$ indicate the u,v frequency selective DCT filter with kernel size f×f. The feature map $F_{m}$ at depth *d* is represented as a weighted linear aggregation of DCT coefficients on all input channels *C* as follows:

(2) $$F_{m}^{d}=\sum_{n=0}^{C-1}\sum_{u=0}^{f-1}\sum_{v=0}^{f-1}w_{n,u,v}^{d}\gamma_{\left(u,v\right)}**F_{m(n)}^{d-1},$$

where $w_{n,u,v}^{d}$ represented the learned weight for the nth feature at frequency *u*, *v* and ∗∗ denoted as the 2-D convolution operator. The transformation process allows the input feature to undergo harmonic decomposition, allowing learned weights to be used for combining the transformed signals.

### 2.4. Harmonic Attention Block

**Figure 3:**
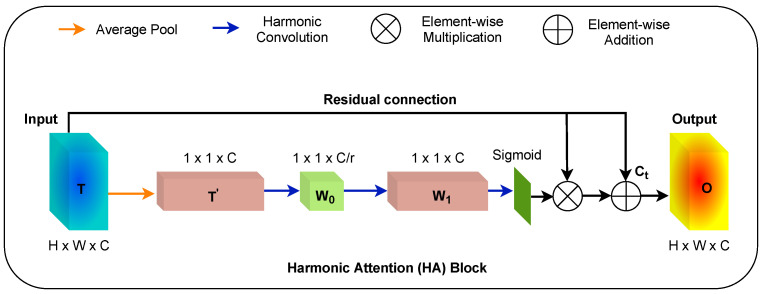
Illustration of harmonic attention (HA) block.

To advance the feature discriminability between the small targeted region and background pixels, Figure 3 presents the proposed harmonic attention (HA) block details. The block makes use of harmonic convolutions to extract feature maps. It is designed to promote feature discriminability between the target objects and their background, i.e., ovary and follicles, in our application. In this block, an input feature map *T* ∈ $R^{C\times H\times W}$ is average pooled to aggregate *C*, which is the channel statistics. This generates $T^{\prime }$ ∈ ${R^{\prime }}^{C^{\prime }\times 1\times 1}$ which is then passed to the two 1×1 harmonic convolution layers to extract non-linear inter-channel relationships with the help of spectral DCT filters. If $W_{0}$ ∈ $R^{C\times \frac{C}{r}}$ and $W_{1}$ ∈ $R^{C\times \frac{C}{r}}$ are weights of two harmonic convolutional layers (where *r* refers to the reduction ratio), then the channel attention map can be formulated as:

(3) $$attn\left(T^{{}^{\prime }}\right)=\sigma (W_{1}*\left(ReLU\left(W_{0}*T^{{}^{\prime }}\right)\right)),$$

where σ(.) is the sigmoid activation function. Finally, the channel attention map can be generated as follows:

(4) $$C_{t}=T*T^{{}^{\prime }}.$$

The use of skip connections between the input feature map *T* and $C_{t}$ helps to narrow the semantic gap and results in the final output map *O*:

(5) $$O=C_{t}+T.$$

### 2.5. Cost Function

We use a weighted sum of the binary cross-entropy (BCE) and focal loss $L^{F}$ which are defined as:

(6) $$\begin{array}{c} L^{F}\left(y,\hat{y}\right)=-y{\left(1-\hat{y}\right)}^{\gamma }\cdot log\left(\hat{y}\right)-\left(1-y\right){\hat{y}}^{\gamma }\cdot log\left(1-\hat{y}\right). \end{array}$$

(7) $$\begin{array}{cc} L^{\mathrm{BCE}}\left(y,\hat{y}\right)= & -(y\cdot log\left(\hat{y}\right)+\left(1-y\right)\cdot log\left(1-\hat{y}\right)), \end{array}$$

The final loss is expressed as follows:

(8) $$Loss_{final}=L^{\mathrm{BCE}}\left(y,\hat{y}\right)+\eta *L^{F}\left(y,\hat{y}\right),$$

where, *y* is the target binary mask, and $\hat{y}$ is the predicted mask obtained by the segmentation model. We used η equal to 0.6 as an empirical weighting factor.

### 2.6. Follicle Counting

**Figure 4:**
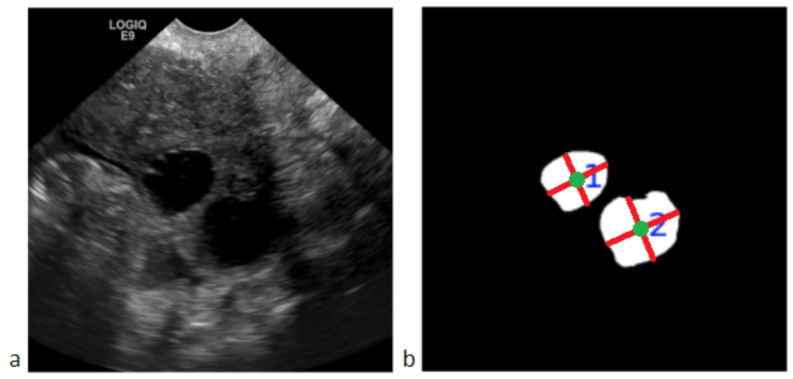
(**a**) shows an ovarian US image with two oval-shaped follicles; (**b**) shows the corresponding follicle segmentation mask. The follicle delineations are white with green dots denoting individual follicles’ centroids. These act as the starting point for calculating the major and minor axis lengths shown in red lines. The diameter is then calculated by multiplying the axis lengths by the pixel size in mm to obtain physical measurements. The follicle count is labeled in blue.

In the third stage, segmented follicles are used to measure the follicle size. Figure 4 shows an ovarian US image with oval-shaped follicles and its corresponding follicle segmentation mask generated by stage 2. The main steps of follicle counting are explained as follows:

**Input:** Single ground truth follicle segmentation mask and predicted follicle segmentation mask.

- Load ground truth mask and predicted mask images.
- Measure follicle diameters on both images in pixels.
- Convert pixel diameters to physical measurements.
- Exclude follicles sized outside the recruitable range of 2–10 mm in diameter by converting pixels black, as counting antral follicles < 2 mm in diameter might heighten the chances of counting small anechoic structures like vessels or artifacts; whereas counting dominant follicles > 10 mm lack the evidence of clinical practicality [3].
- Compute the dice similarity coefficient (DSC) between the ground truth mask and predicted mask images.
- Calculate the number of correctly detected follicles from the predicted mask (follicles with >0.5 Dice coefficient score are considered).
- Calculate the number of detected follicles from the predicted mask.
- Calculate the number of actual follicles from the ground truth mask.
- Evaluate the precision and recall of our predicted follicle counting with the formula from [37].

## 3. Experimental Design and Results

### 3.1. Implementation Details

All the methods are implemented in PyTorch [38]. We use an NVIDIA GeForce RTX 2080Ti GPU with 11 GB RAM. Table 2 summarizes the hyperparameters used to build the proposed segmentation model. The images are resized to 384×384, and the pixel values are normalized between 0 and 1. The training dataset is augmented with random 15-degree rotations and horizontal flips. An ADAM optimizer with $\beta_{1}$= 0.5, $\beta_{2}$ = 0.999, and an initial learning rate of 0.0002 is used to optimize the model better. Step decay learning is activated if the Dice coefficient score for the validation set plateaus for two consecutive epochs. We used a batch size of four images and trained the model for 50 epochs since the model was optimized completely.

This paper organizes our experiments into three key stages: 1. ovary segmentation, 2. follicle segmentation, and 3. follicle counting. To measure the effectiveness of the proposed model on segmentation stages, five evaluation metrics are used, i.e., accuracy, dice similarity coefficient (Dice), intersection over union (IoU), sensitivity, and specificity [39]. The precision and recall metrics are used to evaluate the follicle counts.

### 3.2. Ovary Segmentation

Table 3 demonstrates the quantitative result of proposed HaTU-Net (i.e., Baseline (BL) plus harmonic attention block) compared to five state-of-the-art segmentation methods including U-Net [15], Attention U-Net [17], R2U-Net [34], U-Net++ [35], and DeepLabv3+ [18]. We also demonstrate the result of the Baseline (BL) method consisting of a standard U-Net network with harmonic convolution without an attention block. Experimental results confirm that the HaTU-Net performed significantly better than the second-highest U-Net method in DSC, and IoU metrics with 2%, and 3%, respectively. We observed that the DCT-based spectral kernel aids in learning shape, boundary, and texture mapping from noisy ultrasound images. These noisy images include poor contrast, shadows, speckle variation, and poor signal-to-noise ratio. To reach the optimal version of the proposed method, we performed an ablation study to determine and quantify the perceptiveness of each employed block to segmentation results.

We define our baseline model as a U-Net where it replaces the standard 2D convolutions layers with harmonic convolutions. Since the ovary occupies a large part of an ultrasound image, we enforce fine boundary segmentation while retaining shape information through an HA block (BL + HA). Therefore, adding the harmonic attention block to BL leads to better segmentation results. Finally, HaTU-Net improves the DSC and IoU scores by approximately 1.5% in both metrics compared to our BL model. The characteristics of the attention mechanism allow highlighting the most relevant feature of the hypoechoic ovary region and ignoring the background or acoustic shadows in the US images.

**Figure 5:**
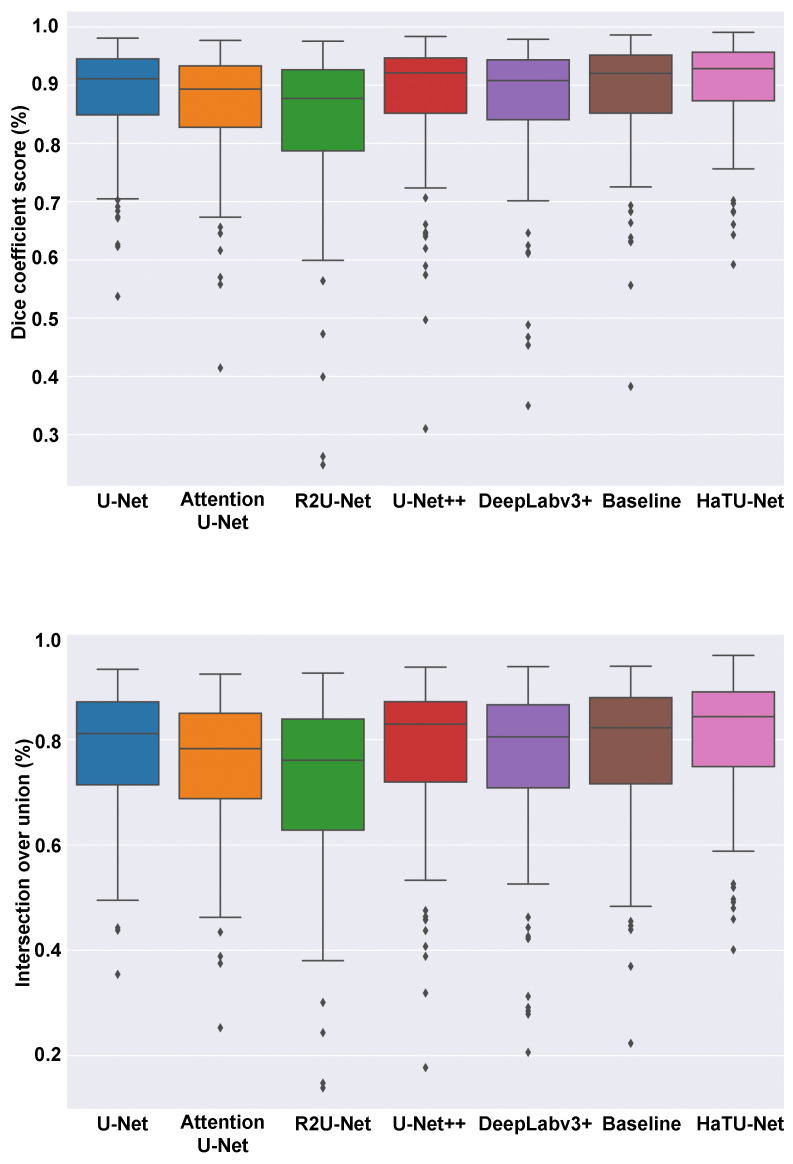
Boxplots of Dice and IoU scores of ovary segmentation.

Figure 5 shows the box-plot analysis of Dice and IoU scores on the ovarian dataset. The HaTU-Net has generated fewer outliers compared to other segmentation methods. For instance, we can see that the proposed method has a lower standard deviation than other methods, showcasing its robustness.

**Figure 6:**
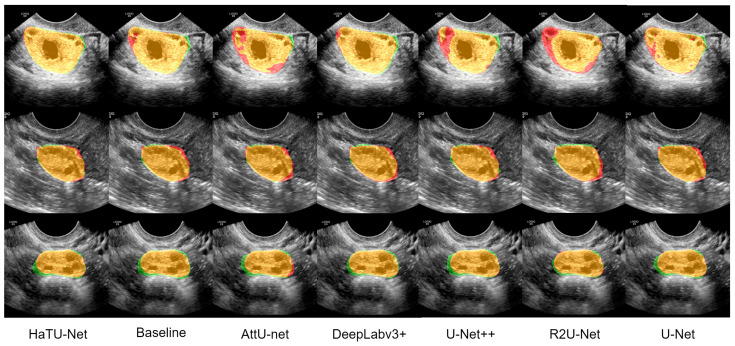
Illustration of three qualitative example results generated by HaTU-Net against five state-of-the-art methods for ovary segmentation.

Further, qualitative analysis plays a crucial role in visually determining segmentation results. Figure 6 exhibits the three qualitative examples results generated by state-of-the-art segmentation methods compared to the HaTU-Net. We provide the color maps that help to identify the true positive (orange), false positive (green), false negative (red), and true negative, including the background. Visual inspection confirms that HaTU-Net precisely segments the ovary boundaries, whereas other methods have produced many false positives.

### 3.3. Follicle Segmentation

**Figure 7:**
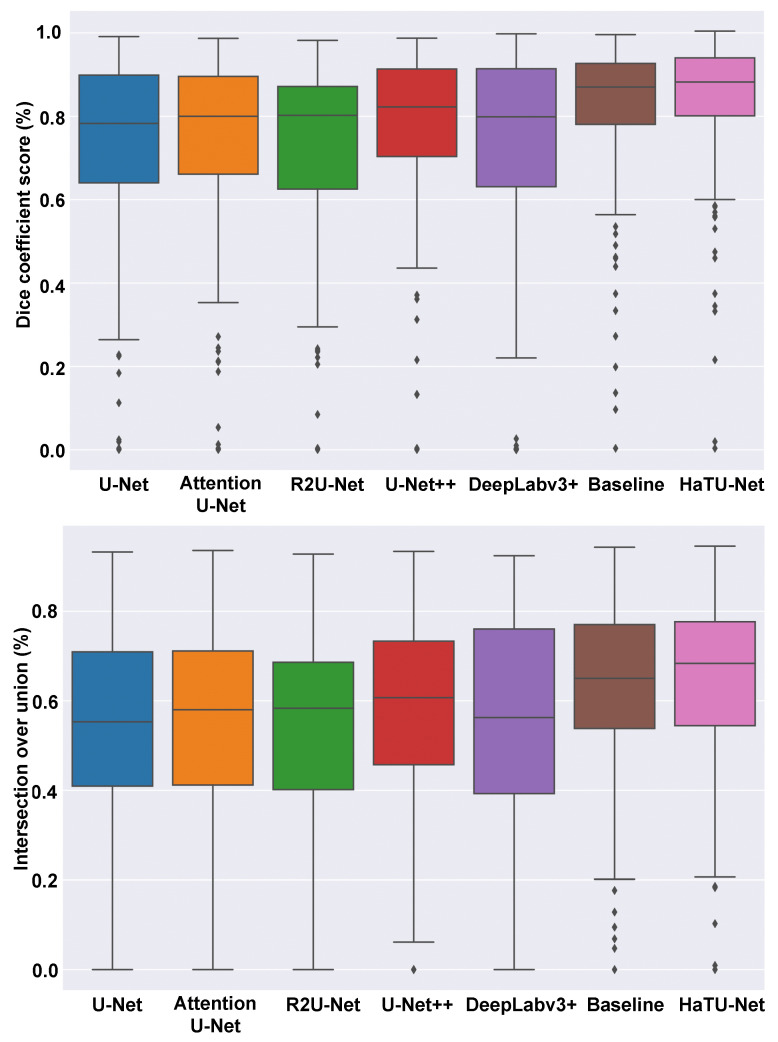
Boxplots of Dice and IoU scores of follicle segmentation.

Table 4 exhibits the follicle segmentation results. The proposed HaTU-Net improved follicle segmentation results with 10% DSC and IoU scores compared to U-Net. Our BL methods demonstrated more remarkable results than other state-of-the-art segmentation methods. We observed that incorporating the HA block leads to better segmentation results. Especially, HA block can capture many small follicles’ shape features and provide separation between them. This allows delineating of the follicle boundaries efficiently. Furthermore, Figure 7 displays the boxplot analysis of Dice and IoU scores. The results confirm that HaTU-Net achieves significantly high mean segmentation results with a lower standard deviation. Additionally, it is seen that it produces fewer outliers than other methods. Note that all values outside the whiskers are considered outliers.

**Figure 8:**
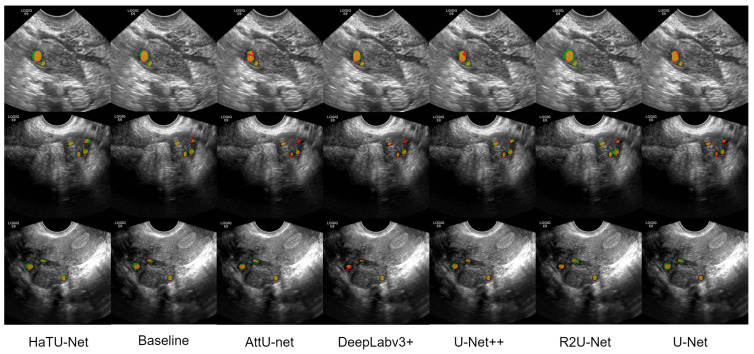
Illustration of three qualitative example results generated by HaTU-Net against five state-of-the-art methods for follicle segmentation.

Figure 8 represents the three examples for qualitative assessment of follicle segmentation. The harmonic attention block helps in refining the boundaries between follicles, as shown by the performance of HaTU-Net in separating follicles for automating the follicle count process.

### 3.4. Ablation Study

Table 5 demonstrates an ablation study to estimate the effect of loss function employing the proposed HaTU-Net method. Our experiments use various combinations of loss functions such as BCE, Dice loss, BCE+Dice, and BCE+Focal loss. BCE+Focal loss leads to 1% refinement over Dice loss in segmentation results. This combination of loss functions focuses on boundaries. Separately, employing the Dice loss gains better results versus BCE+Dice loss. On the follicle dataset, we also see that BCE+ Focal loss yields a 7% and 2% increment in DSC and IoU scores, respectively, compared to BCE+Dice loss. This suggested loss function help detach follicles that appear joined together, thus improving clinical outcome. However, employing only BCE leads to poor segmentation results achieving a 68.10% Dice score.

### 3.5. Follicle Counting

Table 6 confirms the results for follicle counting. Experimental results prove that HaTU-Net attained a very high rate of follicle counting (i.e., precision) against other state-of-the-art models. In addition, we observed that the 76.79% of HaTU-Net gained a 1.5% recall rate compared to R2U-Net. The HaTU-Net helps lower the false positive rate from segmentation artifacts, leading to higher follicle counting.

### 3.6. Discussion and Limitations

We have proposed an efficient segmentation model to segment the ovary and follicle in TVUS images. For this purpose, we replaced the standard convolutional filter using harmonic convolution. The input image was first decomposed using the discrete cosine transform (DCT), and these transformed signals merged using learned weights. We utilized the proposed harmonic attention block that provides a better feature representation of the targeted region (i.e., ovary and follicle) by ignoring the unwanted features that could infer the segmentation performance. Although the existing methods achieved acceptable results, they failed to capture the precise boundaries of the ovary and follicles from noisy US images. We found that the proposed model ignored the imaging artifacts in the presence of shadows, speckle noise, and poor contrast images, leading to better segmentation results than existing approaches. It also produced fewer false-positive pixels and provided a more robust segmentation performance with less error rate. However, our proposed model has the limitation of poorly segmenting the ambiguous boundaries of ovaries and follicles.

## 4. Conclusions

In this paper, we proposed an automated solution for ovarian ultrasound quantification. We have developed a novel method named HaT-UNet that provides accurate segmentation of ovaries and follicles from TVUS images. The proposed HaTU-Net employed harmonic convolution with discrete cosine transform (DCT) and enhanced feature discriminability through HA block to handle ambiguous boundaries. Experimental results proved that the HaTU-Net tackles the presence of imaging artifacts by achieving DSC score improvement of 2% and 10% for the ovary and follicles, respectively, compared to U-Net. Further, the proposed model verified its effectiveness in follicle counting and attained a recall of 91% and a precision of 76.69%. Conclusively, the experimental output demonstrated HaTU-Net’s outstanding ability and provided efficient segmentation results by outperforming other state-of-the-art algorithms. In the future, we will extend the potential of the proposed model to 3D segmentation tasks. Future research could investigate the proposed model’s ability on additional biomedical datasets.

## Figures and Tables

**Table 1:** TVUS ovarian dataset split into the three subsets.

Dataset	Subset	Number of Images
TVUS ovary	Train	466
	Validation	166
	Test	141

**Table 2:** A summary of hyperparameters.

Hyperparameter	Value
Input image size	384×384
Pixel value normalize	0–1
Learning rate	0.0002
Adam optimizer	$\beta_{1}$ = 0.5, $\beta_{2}$ = 0.999
Epochs	50
Batch size	4
Data augmentation	rotation 15 degree and horizontal flipping

**Table 3:** Comparing of the proposed model for ovary segmentation with five state-of-the-art segmentation methods. Statistically significant results are highlighted in bold.

Methods	Accuracy	Dice	IoU	Sensitivity	Specificity
U-Net [15]	96.89±0.02	87.89±0.08	77.54±0.12	90.51±0.09	98.27±0.01
Attention U-Net [17]	96.41±0.02	86.02±0.09	74.89±0.13	87.96±0.11	98.16±0.02
R2U-Net [34]	95.73±0.03	83.31±0.12	71.49±0.15	86.31±0.13	97.53±0.02
U-Net++ [35]	97.14±0.02	87.78±0.10	77.72±0.13	89.42±0.13	98.19±0.01
DeepLabv3+ [18]	96.99±0.02	86.66±0.11	76.21±0.14	87.35±0.15	98.25±0.01
Baseline	97.26±0.01	88.37±0.09	78.51±0.13	90.46±0.12	98.18±0.01
**HaTU-Net**	97.55±0.01	90.01±0.07	80.72±0.11	90.86±0.10	98.57±0.01

**Table 4:** Comparing of the proposed model for follicles segmentation with five state-of-the-art segmentation methods. Statistically significant results are highlighted in bold.

Methods	Accuracy	Dice	IoU	Sensitivity	Specificity
U-Net [15]	99.20±0.01	71.77±0.22	54.18±0.23	74.55±0.25	99.51±0.01
Attention U-Net [17]	99.28±0.01	73.23±0.22	55.56±0.22	73.92±0.24	99.62±0.01
R2U-Net [34]	98.99±0.01	73.28±0.2	56.07±0.21	84.91±0.19	99.17±0.01
U-Net++ [35]	99.35±0.01	75.48±0.20	57.61±0.22	75.58±0.23	99.68±0.01
DeepLabv3+ [18]	99.35±0.01	69.37±0.26	52.36±0.26	66.43±0.28	99.77±0.01
Baseline	99.45±0.01	79.80±0.19	62.51±0.21	81.64±0.20	99.70±0.01
**HaTU-Net**	99.51±0.01	81.40±0.18	64.11±0.20	82.24±0.19	99.78±0.01

**Table 5:** Ablation study of the loss function. Statistically significant results are highlighted in bold.

Dataset	Loss Function	Accuracy	Dice	IoU	Sensitivity	Specificity
Ovary	BCE	97.67±0.01	89.28±0.10	80.06±0.13	88.94±0.14	98.86±0.01
	Dice Loss	97.46±0.02	89.45±0.08	79.97±0.12	89.69±0.12	98.7±0.01
	BCE + Dice	97.44±0.0	89.13±0.09	79.64±0.13	90.76±0.12	98.45±0.01
	BCE + Focal	97.55±0.01	90.01±0.07	80.72±0.11	90.86±0.10	98.57±0.01
Follicle	BCE	99.11±0.00	68.1±0.22	55.6±0.23	62.95±0.26	99.76±0.01
	Dice Loss	99.14±0.01	72.72±0.19	60.26±0.21	72.02±0.20	99.71±0.01
	BCE + Dice	99.18±0.01	74.56±0.18	62.4±0.20	77.44±0.19	99.64±0.0
	BCE + Focal	99.51±0.01	81.40±0.18	64.11±0.20	82.24±0.19	99.76±0.01

**Table 6:** Follicles counting results in five state-of-the-art segmentation methods.

Methods	U-Net	Attention U-Net	R2U-Net	U-Net++	DeepLabv3+	HaTU-Net
Total No. of Images	141					
No. of Real Follicles	378					
No. of Detected Follicles	447	438	482	453	488	448
No. of Correctly Detected Follicles	315	303	350	339	328	344
Precision (%)	70.47	69.18	72.61	74.83	67.21	**76.69**
Recall (%)	83.33	80.16	90.59	89.68	86.77	**91.01**

## Data Availability

The raw/processed data required to reproduce the above findings cannot be shared at this time due to legal/ ethical reasons at our institution.
